# Characterization of dolutegravir drug resistance in persons diagnosed with HIV after exposure to long-acting injectable cabotegravir for preexposure prophylaxis

**DOI:** 10.1097/QAD.0000000000003322

**Published:** 2022-10-03

**Authors:** Amrit Kaur Ahluwalia, Seth Inzaule, Rachel Clare Baggaley, Marco Vitoria, Robin Schaefer, Heather-Marie Ann Schmidt, Michelle Rodolph, Amalia Giron, Michael R. Jordan

**Affiliations:** a Tufts University School of Medicine, Boston, MA, USA; b Global HIV, Hepatitis and Sexually Transmitted Infections Programmes, World Health Organization, Geneva, Switzerland; c UNAIDS Regional Office for Asia and the Pacific, Bangkok, Thailand.

Dolutegravir (DTG) is the cornerstone of the WHO's recommended first-line antiretroviral treatment (ART) regimen, tenofovir disoproxil fumarate + lamivudine + DTG, known as TLD [1]. Expanded use of TLD has led to improvement in population-level HIV viral suppression raising hopes for attaining the 2030 global goal for HIV epidemic control [2].

In phase 3 clinical trials, long-acting injectable cabotegravir (CAB-LA), an integrase strand-transfer inhibitor (INSTI) and analogue of DTG, was shown to be highly efficacious at reducing risk of HIV infection when used for HIV preexposure prophylaxis (PrEP) compared with the oral-PrEP regimen tenofovir-emtricitabine (TDF/FTC) [3,4]. With approval of the US Food and Drug Administration and anticipated approval in other countries, concerns for emergence of drug-resistant HIV because of rollout of CAB-LA for PrEP, which may negatively impact outcomes of DTG-based treatment, have been raised. We sought to characterize the prevalence and patterns of mutations associated with DTG resistance in persons diagnosed with HIV after receiving CAB-LA PrEP.

We conducted a systematic review of available English language literature on PubMed, according to the following search strategy: ‘HIV’ and (resistance) and (’GSK1265744’ or ‘Cabotegravir’). We also searched peer-reviewed abstracts from major HIV conferences between 2019 and 2022. Searches were conducted in March 2022. Bibliographies of relevant articles were screened for additional references. The main analysis included studies reporting resistance emerging from CAB-LA PrEP and excluded pharmacokinetic/pharmacodynamic studies and preclinical trials.

Sixty-nine potentially relevant citations were identified but only two clinical trials met the inclusion criteria and were included in the systematic review. Drug resistance mutations were abstracted and re-analyzed using the Stanford HIV Drug Resistance Database V9.2 (https://hivdb.stanford.edu; accessed on 08 April 2022). DTG and CAB resistance were predicted if mutations or mutation profiles had penalty scores at least 15. A pooled prevalence was estimated using a fixed effects model, while heterogeneity was assessed using the *I*^2^ statistic.

The two studies included a total of 4097 participants receiving CAB-LA, 20 (0.49%) of whom were diagnosed with HIV infection [3–6]. Seven of 20 had predicted CAB and DTG resistance (Table 1). The pooled prevalence of DTG resistance among those infected with HIV while receiving CAB-LA PrEP was 24.4% [95% confidence interval (CI) = 8.1–40.7]; *I*^2^ 85.4 (*P* = 0.009).

**Table 1:** HIV drug resistance-associated mutations and predicted cabotegravir and dolutegravir resistance in people diagnosed with HIV during HIV preexposure prophylaxis with long-acting cabotegravir

*N*	DRM patterns	Predicted cabotegravir resistance	Predicted dolutegravir resistance
1	E138K, Q148K	High	Intermediate
2	L74I, E138E/K, G140G/S, Q148R	High	High
3	E138A, Q148R	High	Intermediate
4	R263K	Intermediate	Intermediate
5	G140A, Q148R	High	Intermediate
6	N155H, R263K, Q148R	High	Intermediate
7	N155H, S230R	Intermediate	Intermediate

Predicted HIV drug resistance was assessed using Stanford HIV Drug Resistance Database v 9.2. DTG and CAB resistance was predicted if the mutations or mutation profiles had penalty scores at least 15. DRM, drug resistance mutations; *N*, number.

Findings indicate that, while the overall number of people testing positive for HIV following CAB-LA PrEP is extremely low, approximately one in four persons diagnosed with HIV carried a virus resistant to DTG. The prevalence of drug resistance observed in this analysis is consistent with estimates reported in CAB treatment trials 32.5% (95% CI = 13.7–51.4) [7–11] (data not shown) and observations from a macaque study designed to evaluate resistance emerging in CAB-LA PrEP [12].

Although there remains uncertainty about treatment outcomes in people testing HIV-positive after receiving CAB-LA if initiated on TLD, alternative treatment options in the context of CAB-LA PrEP may be needed. It is, however, noteworthy that 75% of patients initiating treatment after CAB-LA PrEP are likely to have DTG-susceptible virus. Although pretreatment resistance testing is not recommended under the public health approach, it may be cost-effective for the few patients initiating treatment after CAB-LA exposure and could preserve future treatment options. In the absence of drug resistance testing or alternatives, it may be prudent to initiate patients on DTG-based ART with close treatment monitoring to ensure prompt switch to second-line regimens in cases of virological failure.

Additional research is needed on the feasibility, benefits, and cost-effectiveness of alternative HIV-testing approaches, including using nucleic acid-based HIV tests prior to PrEP initiation and during PrEP to optimize early diagnosis of HIV thereby limiting selection and accumulation of INSTI drug resistance mutations [5]. Critical will be studies assessing how people take CAB-LA PrEP in real-world settings and if more breakthrough infections are observed compared with clinical trials. Of equal importance will be routine HIV drug resistance surveillance in populations receiving PrEP.

The small number of eligible studies and resulting small number of participants contributing data limit the generalizability of the results of this review. Moreover, the frequency and methods of HIV diagnosis and viral genotyping varied across the trials making it difficult to estimate and generalize findings regarding the timings of HIV and drug resistance acquisition [3–6].

In conclusion, while CAB-LA PrEP is highly efficacious, nearly one in four persons diagnosed with HIV after receiving CAB-LA may have DTG resistance prior to treatment initiation. Although concerns over drug resistance should not be a reason to limit CAB-LA PrEP use, findings suggest a need to plan alternative treatment options, and wherever feasible, consider use of drug resistance testing to guide regimen selection, and implement measures to minimize emergence of drug resistance during CAB-LA PrEP.

## Acknowledgements

This work was supported by WHO. Amrit Kaur Ahluwalia was supported by a grant from The Shader Family Fellowship from Tufts University School of Medicine and a Grant for Emerging Researchers/Clinician Mentorship (GERM) from the Infectious Diseases Society of America (IDSA).

### Conflicts of interest

There are no conflicts of interest.
